# Muscular performance characterization in athletes: a new perspective on isokinetic variables

**DOI:** 10.1590/bjpt-rbf.2014.0047

**Published:** 2014

**Authors:** Giovanna M. Amaral, Hellen V. R. Marinho, Juliana M. Ocarino, Paula L. P. Silva, Thales R. de Souza, Sérgio T. Fonseca

**Affiliations:** 1 Programa de Pós-graduação em Ciências da Reabilitação, Universidade Federal de Minas Gerais (UFMG), Belo Horizonte, MG, Brazil; 2 Universidade Estadual de Montes Claros (UNIMONTES), Montes Claros, MG, Brazil

**Keywords:** physical therapy, muscle strength dynamometer, knee joint, isokinetics, factor analysis

## Abstract

**Background:**

Isokinetic dynamometry allows the measurement of several variables related to muscular performance, many of which are seldom used, while others are redundantly applied to the characterization of muscle function.

**Objectives:**

The present study aimed to establish the particular features of muscle function that are captured by the variables currently included in isokinetic assessment and to determine which variables best represent these features in order to achieve a more objective interpretation of muscular performance.

**Method:**

This study included 235 male athletes. They performed isokinetic tests of concentric knee flexion and extension of the dominant leg at a velocity of 60º/s. An exploratory factor analysis was performed.

**Results:**

The findings demonstrated that isokinetic variables can characterize more than muscle torque production and pointed to the presence of 5 factors that enabled the characterization of muscular performance according to 5 different domains or constructs.

**Conclusions:**

The constructs can be described by torque generation capacity; variation of the torque generation capacity along repetitions; movement deceleration capacity; mechanical/physiological factors of torque generation; and acceleration capacity (torque development). Fewer than eight out of sixteen variables are enough to characterize these five constructs. Our results suggest that these variables and these 5 domains may lead to a more systematic and optimized interpretation of isokinetic assessments.

## Introduction

Over the last decades, the technology of isokinetic devices has improved^1^^,^ 2. In order to achieve a more thorough description of muscular performance, new variables began to be calculated and included in the assessment reports generated by these devices^1^. However, only a few of these variables have been explored from scientific and clinical perspectives. For example, peak torque has been the most widely reported and discussed approach to the characterization of muscular performance for several years^1^^,^ 3 ^,^ 4.

Isokinetic assessments of muscle function are widely used to identify specific deficits, or to assess the results of interventions. Some authors have discussed the relevance and meaning of each variable included in isokinetic assessments^1^^,^ 4 ^-^ 6. Some publications reported on variables such as Total Work, Fatigue Index and Power, in addition to Peak Torque^6^^,^ 7. However, little is known about the associations among such variables, as well as the individual contribution of each variable to the characterization of muscular performance. A better understanding of these aspects might help to establish which variables measure similar features of muscle function and which variables best represent particular features of performance. Such understanding would allow the report of variables in a uniform and rational manner. Therefore, the aims of the present study were to identify the specific features of muscle function that are represented by the variables currently available in isokinetic assessments and to determine which variables best represent these features in order to develop a more objective assessment of muscular performance.

## Method

### Subjects

Preseason isokinetic assessment reports of knee joint flexion-extension concentric motions at 60º/s were selected from the laboratory's database. The reports showing a documented history of lower limb injury or symptoms were excluded and only data from the dominant limb were included. The sample included 235 male elite athletes (soccer and volleyball players) with mean age 23.07±4.84 years, mean height 1.83±8.09 meters and mean body weight 78.18±9.33 Kg. All athletes were, at the time of the evaluation, active in their professional team. The present study was approved by the Ethics Committee of Universidade Federal de Minas Gerais (UFMG), Belo Horizonte, MG, Brazil (approval number 01748412.0.0000.5149), and all athletes signed an informed consent form.

### Procedures

The procedure was explained and the lower limb dominance was determined by asking the athlete which leg he uses to kick a ball. The athletes performed a warm-up consisting of exercises on an ergometric bicycle for 5 minutes. Next, the athletes were placed on the isokinetic dynamometer (Biodex Multi-joint System 3, Biodex Medical Systems Inc, Shirley, NY, USA) in a sitting position with hip flexion of 85Âº and the equipment axis aligned with the lateral condyle of the femur. The arms were placed along the sides of the body, the trunk was stabilized against the backrest using the chair belts, the thigh of the tested limb was fixed against the seat by means of a belt, and the contralateral limb was allowed to hang free. The tested leg was weighted to correct for the effects of gravity on the torque measured, according to the specifications of the Biodex Manual. To assess muscular performance, the participants were asked to perform alternating concentric contractions of the knee flexors and extensors within a range of motion of 85Âº (90Âº to 5Âº of flexion). During the test, the participants were instructed to keep the maximum force throughout the entire range of motion. In addition, they were encouraged to go faster and never stop until the end of the assessment. The participants were allowed to familiarize themselves with the procedures before actual testing by performing 3 repetitions of the tested motion. Then they performed a set of 5 repetitions at 60Âº/s. When the Coefficient of Variation (CV) of the Peak Torque was higher than 10%^8^, the athlete was allowed to rest and the set was repeated.

### Variables selection

Sixteen variables available in the Comprehensive Evaluation Reports generated by Biodex Software were selected to be included in this study: Peak Torque, Time to Peak Torque, Angle of Peak Torque, Torque at 30Âº, Torque at 0.18 s, Coefficient of Variation, Maximum Work, Maximum Work Repetition Number, Total Work, Work Last Third, Work First Third, Work Fatigue Percentage, Average Power, Acceleration Time, Deceleration Time, and Average Peak Torque. The windowing option was turned on to guarantee that only the isokinetic portion (above 70% of the preset speed) of the test was used. Peak Torque and Maximum Work normalized by bodyweight were not included in the analysis, since normalization would make the results dependent on the individual's mass. Another variable not used in the present study was the Agonist:antagonist ratio, as it does not pertain to the assessment of a specific muscle group.

### Statistical analysis

The present study used an exploratory factor analysis to identify the factors that could accurately characterize muscular performance. This approach assumes the presence of associations and redundancy among the variables included in the isokinetic report. Factor analysis is a set of statistical techniques used to explain the relationship between original observed variables and non-observed variables (factors). Therefore, the number of factors identified is lower than the number of original variables analyzed. Each factor characterizes one theoretical aspect (construct) of muscular performance.

Initial exploratory factor analysis with varimax rotation was performed with the SPSS 15.0 statistical software (SPSS Inc., Chicago, IL, USA). The factors that exhibited an eigenvalue >1 were maintained. The Kaiser-Meyer-Olkin (KMO) measure of sampling adequacy and Bartlett's test of sphericity were run to confirm the adequacy of factor analysis. The variables with communality values (proportion of common variance) lower than 0.6, as well as those with cross loadings over 0.4, were excluded from the analysis. These variables were excluded successively, and a new factor analysis was performed following the removal of each variable until the goodness-of-fit of the reduced model was attained.

In order to identify outliers for each factor of the reduced model, regression scores were computed for each individual. Following the removal of the outliers in these scores, the final exploratory factor analysis was performed. To validate the model relative to the knee extension torque curve data, the sample was randomly divided into 2 subsamples ("split-sample" method), and factor analysis was performed in each subsample to assess whether the initial factor structure was maintained. Finally, to investigate the capacity of generalization of the final factor structure, a second exploratory factor analysis, which included all the variables used in the first analysis, was performed using the knee flexion torque curve data. The similarity between the factor structures generated based on the knee extension and flexion data was assessed by means of Tucker's congruence coefficient.

## Results

Upon initial exploratory analysis (n=235), Bartlett's test of sphericity was significant (p<0.0001), and the KMO measure of sampling adequacy was 0.700, which indicated that factor analysis was appropriate for the data in the present study. These results pointed to the presence of 5 factors that clearly represented different features of muscular performance, and we chose to maintain this initial (5-factor) structure in the subsequent analyses (Table 1).

**Table 1:** Factor structure of knee extensor isokinetic assessment data disclosed by the initial exploratory factor analysis.

Factors						
Variables	1	2	3	4	5	Communality
Maximum Work	0.976	0.042	0.110	–0.071	–0.073	0.977
Total Work	0.961	0.042	0.036	0.005	–0.123	0.943
Work Last Third	0.944	0.062	–0.163	–0.137	–0.106	0.952
Work First Third	0.929	0.037	0.260	0.025	–0.155	0.957
Peak Torque	0.883	–0.156	–0.023	–0.081	0.354	0.937
Average Peak Torque	0.865	–0.190	–0.138	–0.072	0.337	0.922
Average Power	0.787	–0.216	–0.150	–0.156	0.421**	0.890
Acceleration Time	0.082	0.865	–0.020	0.098	0.277	0.841
Torque at 0.18 s	0.400**	–0.824	–0.049	0.030	0.290	0.927
Time to Peak Torque	0.113	0.669	0.143	–0.389	–0.476**	0.859
Work Fatigue Percentage	–0.101	–0.052	0.795	0.312	–0.089	0.750
Maximum Work Repetition Number	–0.035	–0.019	–0.749	0.167	0.230	0.643
Coefficient of variation	0.045	0.087	0.696	0.071	0.149	0.522*
Angle of Peak Torque	–0.004	–0.107	0.140	0.940	0.027	0.916
Torque at 30º	0.601**	–0.210	–0.035	–0.680	–0.098	0.878
Deceleration Time	–0.033	–0.001	0.045	0.018	–0.830	0.692
**Percentage of Explained Variance (%)**	** 39.6% **	** 12.9% **	** 11.7% **	** 10.6% **	** 10.3% **	

* Communality <0.6

** Cross loading ≥0.4.

Application of the procedures to reduce the number of variables in the model resulted in the exclusion of 5 variables. The Coefficient of Variation was the first variable to be excluded (communality = 0.522). Next, the variables Torque at 30Âº, Time to Peak Torque, Average Power, and Peak Torque at 0.18 s were successively excluded (cross loading >0.4). Following the identification and removal of outliers of the resultant scores (n=219), the reduced model of exploratory factor analysis of the knee extension data explained 90.746% of the total variability of the data. The KMO value was 0.723, and Bartlett's test of sphericity was significant (p<0.0001), indicating that factor analysis was appropriate for the investigated dataset. The variables exhibited adequate communality values (Table 2).

**Table 2:** Factor structure of knee extensor isokinetic assessment data disclosed by the final exploratory factor analysis.

Factors						
Variables	Torque Generation Capacity	Variation in Torque Generation Capacity	Movement Deceleration Capacity	Mechanical/ Physiological Factors of Torque Generation	Acceleration Capacity	Communality
Maximum Work	0.977	0.077	0.060	–0.043	0.017	0.966
Total Work	0.958	0.011	0.093	0.018	–0.005	0.927
Work Last Third	0.935	–0.186	0.078	–0.155	0.022	0.940
Work First Third	0.935	0.258	0.095	0.042	–0.016	0.952
Peak Torque	0.883	–0.095	–0.308	–0.064	0.044	0.889
Average Peak Torque	0.859	–0.172	–0.314	–0.072	0.023	0.871
Work Fatigue Percentage	–0.096	0.812	0.031	0.365	–0.069	0.808
Maximum Work Repetition Number	–0.047	–0.854	–0.074	0.190	–0.108	0.784
Deceleration Time	–0.027	0.073	0.960	–0.023	0.031	0.929
Angle of Peak Torque	–0.073	0.041	–0.020	0.956	–0.024	0.923
Acceleration Time	0.024	0.045	0.028	–0.028	0.995	0.993
**Percentage of Explained Variance (%)**	** 46.9% **	** 14.0% **	** 10.5% **	** 10.2% **	** 9.2% **	

Factor analysis of the 2 randomized subsamples (n1=110, n2=109) exhibited KMO values of 0.698 and 0.683, respectively. Bartlett's test of sphericity was significant (p<0.0001) in both samples. The total explained variance of the data in these subsamples was 91.288% and 90.537%, respectively (Table 3). These 2 analyses converged towards the same structure in the final model, which therefore supported its validation.

**Table 3:** Factor structure of knee extensor isokinetic assessment data disclosed by exploratory factor analysis of the 2 subsamples obtained by means of the "split-sample" method.

	Factors in random sample 1						Factors in random sample 2					
Variables	Torque Generation Capacity	Variation in Torque Generation Capacity	Movement Deceleration Capacity	Mechanical/ Physiological Factors of Torque Generation	Acceleration Capacity	Communality	Torque Generation Capacity	Variation in Torque Generation Capacity	Movement Deceleration Capacity	Mechanical/ Physiological Factors of Torque Generation	Acceleration Capacity	Communality
Maximum Work	0.983	0.041	–0.293	–0.052	0.030	0.974	0.965	0.139	0.047	–0.055	0.022	0.957
Total Work	0.988	0.008	0.056	0.004	–0.006	0.979	0.917	0.045	0.138	0.03	–0.004	0.863
Work Last Third	0.942	0.203	0.108	–0.129	0.021	0.957	0.926	–0.154	0.049	–0.188	0.04	0.920
Work First Third	0.956	–0.199	0.062	0.049	–0.002	0.960	0.899	0.349	0.124	0.017	–0.036	0.947
Peak Torque	0.905	0.041	–0.293	–0.052	0.030	0.911	0.866	–0.167	–0.303	–0.059	0.051	0.875
Average Peak Torque	0.885	0.115	–0.287	–0.057	–0.006	0.883	0.838	–0.243	–0.32	–0.066	0.046	0.870
Work Fatigue Percentage	–0.064	–0.811	–0.104	0.353	–0.065	0.801	–0.141	0.812	0.126	0.349	–0.102	0.828
Maximum Work Repetition Number	–0.012	0.816	–0.127	0.218	–0.137	0.747	–0.101	–0.886	–0.056	0.150	–0.110	0.833
Deceleration Time	–0.050	–0.025	0.963	–0.020	–0.051	0.934	–0.008	0.115	0.949	–0.013	0.100	0.925
Angle of Peak Torque	–0.064	–0.031	–0.009	0.950	0.027	0.908	–0.085	0.056	–0.014	0.967	–0.063	0.949
Acceleration Time	0.017	–0.065	–0.052	0.022	0.989	0.987	0.031	0.027	0.095	–0.068	0.988	0.991
**Percentage of Explained Variance (%)**	** 48.7% **	** 13.0% **	** 10.5% **	** 10.0% **	** 9.2% **		** 44.8% **	** 15.6% **	** 10.6% **	** 10.3% **	** 9.3% **	

In the exploratory factor analysis of the knee flexion data, Bartlett's test of sphericity was also significant (p<0.0001), the KMO value was 0.718, and the explanatory percentage was 91.322% (Table 4). Tucker's congruence coefficient between the flexion model and the final extension model was 0.95, thus indicating high similarity between models. These results demonstrate the capacity of generalization of the final model obtained from the knee extensor isokinetic assessment data to the knee flexor isokinetic assessment data at 60Âº/s.

**Table 4:** Factor structure of knee flexor isokinetic assessment data disclosed by the exploratory factor analysis.

Factors						
Variables	Torque Generation Capacity	Variation in Torque Generation Capacity	Movement Deceleration Capacity	Acceleration Capacity	Mechanical/ Physiological Factors of Torque Generation	Communality
Maximum Work	0.968	0.128	0.091	0.007	0.021	0.962
Total Work	0.977	0.048	0.065	–0.023	0.045	0.963
Work First Third	0.938	0.276	0.027	0.036	0.01	0.959
Work Last Third	0.918	–0.224	0.183	–0.101	0.111	0.948
Peak Torque	0.912	–0.007	–0.126	–0.132	–0.115	0.879
Average Peak Torque	0.912	–0.068	–0.128	–0.16	–0.114	0.891
Work Fatigue Percentage	–0.04	0.845	–0.248	0.157	–0.2	0.841
Maximum Work Repetition Number	–0.113	–0.8	–0.305	0.098	0.056	0.759
Deceleration Time	0.018	0.032	0.938	0.126	–0.036	0.899
Angle of Peak Torque	–0.019	–0.195	–0.04	0.046	0.969	0.981
Acceleration Time	–0.130	0.036	0.122	0.964	0.044	0.964
**Percentage of Explained Variance (%)**	** 48.3% **	** 14.0% **	** 10.3% **	** 9.4% **	** 9.3% **	

## Discussion

The results indicated that the set of variables included in knee isokinetic assessment reports could be represented by 5 factors, which together explained more than 90% of the variance in data. On the one hand, the results indicate much redundancy in the information provided by the variables currently included in isokinetic assessments; on the other, they indicate that 5 different domains of muscular performance are represented by this set of variables. These domains were defined as torque generation capacity, variation in torque generation capacity along repetitions, movement deceleration capacity, mechanical/physiological factors of torque generation, and acceleration capacity (torque development). The identification of these domains should enable a more systematic and optimized interpretation of the data in isokinetic assessments.

Five variables were not included in the final model. The CV had a low communality with the other variables, which is due to the fact that this variable was controlled in our study. The remaining variables (Time to Peak Torque, Torque at 30Âº, Torque at 0.18 s, and Average Power) had cross loading >0.4 for more than one factor (i.e. they bring ambiguous information to test interpretation). For example, Time to Peak Torque and Torque at 30Âº depend on multiple attributes, such as the individual's capacity to produce torque and to accelerate the limb, as well as on muscle length. Thus, the non-inclusion of these variables eliminated redundant information from the test results, as specific aspects of muscle performance were better captured by other variables available in the isokinetic report.

The first factor included the variables that were related to the construct of Torque Generation Capacity. Higher values for these variables were associated with greater torque generation capacity in athletes. This factor captured the largest percentage of the data variability (46.9% of the total variance). The 4 variables that exhibited the greatest factor loading were Maximum Work (0.977), Total Work (0.958), Work First Third (0.935), and Work Last Third (0.935), and these variables also exhibited strong mutual correlation (>0.90). Work, calculated as the area under the force vs. displacement curve, represents the energy spent by muscle exertion during motion (product of torque times angular displacement)^4^^,^ 9 ^,^ 10. Maximum Work represents the capacity to generate muscle torque throughout the full range of the movement repetition that exhibits the greatest muscle work production^4^^,^ 9. Total Work represents the sum of the work calculated for each repetition^9^, and Work First Third and Work Last Third represent the amount of work performed in those stages of movement in all the test repetitions taken together^11^. This factor was also represented by the variables Peak Torque and Average Peak Torque, with factor loading values of 0.883 and 0.859, respectively. Peak Torque represents the maximum torque generated at a single point of the entire range of motion among all test repetitions^9^, whereas Average Peak Torque represents the mean value of the maximum torque generated in all 5 repetitions^11^. The high association between Peak Torque and Average Peak Torque was expected, since only tests with small Coefficient of Variation (<10%) were allowed in this study. When this criterion is not observed and large variation occurs, lower association between these variables can be expected.

The variable Maximum Work best represented torque generation capacity because it exhibited the greatest factor loading for the first factor, in addition to strong correlation with the variables Total Work, Work First Third, and Work Last Third. Although Peak Torque (factor loading of 0.883) has been the variable most widely used in the interpretation of isokinetic assessments, the results of the present study reinforce the need to measure Maximum Work to achieve an accurate characterization of the torque generation capacity. Therefore, the variable Peak Torque should not be used alone to represent that construct, as it could lead to errors in the interpretation of the results. The limited ability of Peak Torque to characterize the torque generation capacity of an individual may be related to the fact that it corresponds to the torque generated at a single point of the entire range of motion. Conversely, the variable Maximum Work provides information on the ability of the muscle to generate torque throughout the entire range of motion^4^. Within that context, individuals able to generate high peak torque do not systematically exhibit the greatest values for Maximum Work^4^. Moreover, high Peak Torque values not associated with Maximum Work values may indicate a condition in which the assessed individual is able to generate high torque at a given point but cannot maintain that level of performance throughout the entire range of motion of the knee joint. The results of the present study therefore suggest that both variables (i.e. Maximum Work and Peak Torque) should be included in reports to achieve a thorough characterization of torque generation capacity related to muscular performance^12^.

The second factor identified was represented by the variables Maximum Work Repetition Number (-0.854) and Work Fatigue Percentage (0.812), which were associated with the Variation in Torque Generation Capacity. This factor contributed 14% of the total explained variance and provided information on the consistency of muscular performance, i.e. the maintenance of torque generation capacity during repetitions. The discrete variable Maximum Work Repetition Number represents the number of the repetition (i.e. 1, 2, 3, 4 or 5) in which the curve exhibited its greatest magnitude^11^. The variable Work Fatigue Percentage represents the percent reduction in the work generated between the first and last thirds of the series of repetitions^13^^,^ 14. For lower scores corresponding to this variable, there is generally greater consistency in muscular performance^13^^,^ 14. However, this analysis must be performed with caution because this variable was calculated based on only 5 repetitions at a velocity of 60Âº/s, and it merely represents the effect of the variation of performance between the beginning and the end of the test. Therefore, in the present study, Work Fatigue Percentage seems to have provided information concerning performance variability. Furthermore, the variable Maximum Work Repetition Number exhibited an inverse correlation with this factor. In other words, the later the Maximum Work repetition occurs, the lower the variability in the response is. Due to the weak correlation between these variables (-0.416), combined use of both may provide information on the ability to maintain the generated torque during repetitions, which is considered to be indicative of muscle endurance or the neuromuscular ability to keep torque generation constant^13^^,^ 14.

The third factor captured the movement deceleration capacity, represented by the variable Deceleration Time, and contributed to 10.5% of the total variance. Deceleration Time represents the total time to reduce isokinetic velocity to 0Âº/s at the end of the motion. During an isokinetic testing, the equipment imposes increasing resistance to any torque that attempts to produce movement speeds greater than that selected for the test. Considering that a proper isokinetic assessment requires that the individual produce maximum torque at any point during the test (resulting in an adequate test speed), the Deceleration Time may characterize the capacity of the individual to maintain maximum torque, at the required speed, close to the end of the tested range of motion (in a position in which the muscle is close to active insufficiency). Thus, greater Deceleration Time values may be associated with lower capacity to maintain torque at the extremes of the range of motion. As this variable represents a different condition in comparison to the other variables, it may add relevant information concerning the isokinetic test^15^. Although it is seldom reported in studies, this variable represents a domain of muscular performance that should not be neglected.

The fourth factor was represented by the Angle of Peak Torque, which corresponded to 10.2% of the total explained variance. This factor was associated with the muscle function domain that we defined as Mechanical/Physiological Factors of Torque Generation. The Angle of Peak Torque corresponds to the position of the joint at the moment when Peak Torque is generated^7^^,^ 16 ^-^ 18. Therefore, this variable represents the optimal point of the torque vs. angular displacement curve for torque development and is related to the interaction between physiological factors such as optimal muscle length (length-tension relationship) and mechanical factors (changes in the angle of insertion/lever arm during rotatory motion) during performance assessment^16^^,^ 18. The interpretation of this variable must take into account not only the absolute values of angulation but also the representation of such angulation relative to the activity of interest. For instance, the Angle of Peak Torque values for the knee flexors and extensors of soccer players were shown to be significantly decreased and increased, respectively, in comparison to cyclists^18^. Furthermore, Angle of Peak Torque that does not meet the specific demands of various sports may be associated with a higher incidence of injuries^17^^,^ 19.

The fifth factor was associated with the muscular performance domain that we termed acceleration capacity. Acceleration time was the only variable that contributed to this factor, and this variable corresponds to the time needed for the limb to reach the velocity pre-established for the isokinetic test when starting from the rest position. Furthermore, this variable may be considered an indicator of an individual's neuromuscular capacity to develop torque quickly^1^^,^ 20 ^-^ 22. Reduced Acceleration Time values may denote superior muscle fiber recruitment capacity of tested muscles and may be associated with shorter latency for torque generation in such muscles^20^^-^ 22. However, the ability to generate high torque values may not suffice to ensure adequate performance, as the speed with which torque is developed must also be taken into account for the characterization of muscular performance. Thus, for a complete assessment, Acceleration Time should be included to address neuromuscular factors related to muscular performance^21^^,^ 22. However, it is important to notice that this variable can be more relevant in test conditions involving higher velocities. In these situations, the acceleration demand is more evident and the acceleration capability is crucial for overall performance in the test.

The aforementioned results were reproduced in the analysis of the 2 randomized subsamples generated from the initial sample, the factor structure found in the analysis of knee extensor isokinetic assessment data was therefore fully validated. In addition, this model was also stable during the analysis of the data resulting from isokinetic assessment of another variety of movement (knee flexion). Although there was an inversion in the distribution for the variables Angle of Peak Torque and Acceleration Time in factors 4 and 5 in the analysis of knee flexor isokinetic assessment data (Table 4), the structure of each factor was maintained (i.e. the way in which these variables were distributed among the different factors remained the same), which may be related to the very similar percentages of variance explained by those factors (i.e. 9.4% and 9.3%, relative to the flexor data). This inversion in the distribution of the variables does not invalidate the structure of the reduced model because the same 5 constructs were still represented. Therefore, muscular performance could be characterized by means of 5 distinct domains.

Factor analysis enabled the identification of 5 different domains that together provided information concerning muscular performance in knee flexion-extension isokinetic assessment at a velocity of 60Âº/s in young athletes. Caution is recommended when generalizing the results for different velocities or populations. The results of the present study point to the relevance of the analysis and the inclusion of variables that represent distinct constructs but are often neglected in the interpretation of isokinetic assessments.

## Conclusions

The present study identified five factors that were accurately represented by only a few variables included in isokinetic reports. Each factor represents a different dimension of muscular performance. Our results suggest that Maximum Work should be systematically reported to characterize torque generation capacity. The constructs movement acceleration and deceleration capacity must be more thoroughly explored in future studies, as they provide different information to that supplied by variables describing torque generation capacity. Finally, variability in torque generation capacity and the contribution of mechanical and physiological factors to torque generation may be accurately represented by variables of Maximum Work Repetition Number, Work Fatigue Percentage, and Angle of Peak Torque. Therefore, the use of just a few variables may suffice to capture the full scope of information provided by isokinetic assessments.
